# 单孔胸腔镜技术培训的思考

**DOI:** 10.3779/j.issn.1009-3419.2018.04.04

**Published:** 2018-04-20

**Authors:** 余明 朱, 格宁 姜

**Affiliations:** 200433 上海，同济大学附属上海市肺科医院胸外科 Department of General Thoracic Surgery, Shanghai Pulmonary Hospital, Tongji University School of Medicine, Shanghai 200433, China

**Keywords:** 单孔电视胸腔镜, 训练, 教育, 微创胸外科, Uniportal video-assisted thoracoscopic surgery, Training, Education, Minimally invasive thoracic surgery

## Abstract

近年来，单孔胸腔镜技术迅速发展，已成为全球外科发展方向。单孔胸腔镜外科医师的针对性，规范化、系统化培训已成为重要的课题，技术培训是保证手术安全性的必不可少的重要环节。单孔电视胸腔镜技术培训应包括：由临床大中心经验丰富的专家直接面对面的教授手术技巧，或聘请专家导师前往学员所在单位进行针对性现场指导，这是一个代表性的、不可缺少的重要环节。网络视频通常也可以作为培训的方式。目前的技术提供了很多模拟训练，诸如：体外模拟器，人工合成胸腔、肺模型，动物实验，3D和VR技术能够为学习者提供各种层面的培训需求。对于大样本量的培训中心，短期的集中培训和中长期的系统性培训目前越来越受关注。根据学员的分级评估，采用多元化的培训模式，因材施教的针对性训练，有助于提高培训效果。我科在单孔胸腔镜外科医师培训方面做了一些工作，积累了一些培训经验，我们认为这样的培训是可行且完全有必要的。

## Abstract (en)

Recently, uniportal video-assisted thoracic surgery (VATS) has developed rapidly and has become the main theme of global surgical development. The specific, standardized and systematic training of this technology has become an important topic. Specific training in the uniportal VATS approach is crucial to ensure safety and radical treatment. Such training approach, including a direct interaction with experienced surgeons in high-volume centers, is crucial and represents an indispensable step. Another form of training that usually occurs after preceptorship is proctorship: an experienced mentor can be invited to a trainee's own center to provide specific on-site tutelage. Videos published online are commonly used as training material. Technology has allowed the use of different models of simulators for training. The most common model is the use of animal wet laboratory training. Other models, however, have been used mostrecently, such as the use of 3D and VR Technology, virtual reality simulators, and completely artificial models of the human thorax with synthetic lung, vessel, airway, and nodal tissues. A short-duration, high-volume, clinical immersion training, and a long term systematic training in high-volume centers are getting more and more attention. According to the evaluation of students' grading, a diversified training mode is adopted and the targeted training in accordance with different students helps to improve the training effect. We have done some work in systematic and standardized training of uniportal VATS in single center. We believe such training is feasible and absolutely necessary.

微创电视胸腔镜（video-assisted thoracic surgery, VATS）技术是当前胸外科最新技术发展的主要方向，与传统开胸手术相比，胸腔镜手术能够减少住院时间、减轻患者术后疼痛、提高患者生活质量^[1]^。随着手术器械的改进、手术医师的熟练程度增加，以及微创化观念深入人心，单孔胸腔镜手术应运而生。2004年，Rocco等^[2]^首先报道了单孔胸腔镜肺楔形切除术，直至2010年，单孔胸腔镜技术在全球范围内迅速发展，并开始应用于肺叶切除术。近年来，腔镜外科手术在越来越多的传统手术领域都获得了革命性的成功，成为全球外科发展的主旋律，追求手术的" 微创" 化成为外科医生的信念和行动，越来越多的肺部复杂手术亦在单孔胸腔镜下完成^[3]^。

由于胸腔镜外科手术的特殊性，胸腔镜外科医师应具备何种资质、如何进行规范化、系统化培训及采用何种方法进行培训就成为重要的课题。在过去的几年里，我科在胸腔镜外科医师系统化规范化培训方面做了一些工作，积累了一些培训经验，取得了较满意的效果，现总结如下，供同行参考借鉴。

## 1 单孔胸腔镜外科医师培训的必要性

胸腔镜手术是通过观察监视器的图像画面来完成手术。单孔胸腔镜通常经一个3 cm-4 cm长，位于第5肋间，不需撑开肋骨的小切口，利用同轴的手术器械和（5 mm或10 mm）观察镜头来完成胸外科诸如肺叶切除术和淋巴结清扫手术的技术^[4, 5]^。与传统多孔VATS相比，由于器械和观察镜平行，类似开胸手术视角。因此，理论上单孔入路可以提供更加灵活自由的视野和器械活动度。

随着技术水平的进步，器械的更新，促使单孔VATS技术已在全球范围内被接收认可。Gonzalez-Rivas等^[6]^最早报道了单孔VATS标准肺叶切除术和系统性淋巴结清扫治疗肺癌。如今，对于一个单孔VATS专家来说，凭借该技术完成全肺切除术，肺叶袖式切除术，血管支气管成型术已成为可能^[7]^。然而，从技术角度，单孔VATS确实存在困难：仅通过一个小切口，凭借电视屏幕的二维平面图像完成胸外科手术，面对突发的术中并发症，以及来自扶镜助手的压力等，这些都严重限制了单孔胸腔镜技术在青年胸外科医生中的开展传播。

一个关键性的问题在于：一项外科技术能否被主流认可，取决于这项技术能否被同行安全的掌握应用^[8]^。因此，为了能保证安全的进行这一手术，全面专业系统性的培训对与要从事单孔VATS外科医师是必不可缺的^[9, 10]^。外科医师的独立性和自信心是贯穿整个手术过程的核心，这也正是需要从规范系统的培训中掌握的。

## 2 最初接触单孔VATS技术时的不适

相比传统胸外科医师，在最初接触单孔VATS技术时会有明显不适应，主要有以下几点。

### 2.1 手眼不配合

由于监视器图像与真正手术部位的距离、方向及与周围脏器的关系不大一致，会产生不适应、判断不准确，操作时就会出现手眼不协调、器械不听指挥现象。

### 2.2 辨认失误和动作不到位

二维平面图像所显示的各组织脏器间的关系不像肉眼直视下的解剖关系那样有立体感，单孔腔镜的视野与传统多孔胸腔镜差别很大，使手术者不能准确地辨认和正确地判断，造成了操作上的困难。

### 2.3 手术中缺乏手感和直视识别

传统外科医师在开腹手术时可以通过手的触摸及肉眼直视来对组织器官及病变进行辨认，而全胸腔镜技术只能靠器械和图像来辨认，给手术带来很大困难。

### 2.4 设备的特殊性

外科医师对各种胸腔镜设备的性能和原理要经过一段时间的学习认识和训练熟悉的过程，才能掌握应用。此外，胸腔镜器械有其特殊性：加长了手术器械的长度，要求操作更具稳定性；不同生产厂家的操作手柄各不相同，器械的精细程度及力度也不同。

### 2.5 操作技术上的不同

传统外科手术无论其难易, 都离不开切开、结扎、缝合、止血四大外科基本技术。胸腔镜的四大基本技术与传统外科有着明显的不同，目前大多胸腔镜手术都借助高能器械平台来完成这些操作，必须靠基本训练及长时间的经验积累。

综上，对于有志于从事单孔VATS手术的胸外科医师，系统全面的培训是必不可少的。

## 3 单孔胸腔镜外科医师培训对象

对于没有普通胸心外科工作基础的初学者，接触学习单孔胸腔镜会遇到较多困难，但也不否认有一部青年医师直接跨过传统多孔VATS培训而直接接收单孔VATS培训。但总体认为具有一定普胸外科或者传统微创胸腔镜工作基础的临床胸外科医师接受单孔VATS培训更具优势。

### 3.1

有普通胸外科经历、有一定胸心血管外科手术基础，熟练掌握传统胸心外科技术微创外科手术是在传统手术的指导原则下，在满足和达到传统手术治疗效果的基础上，通过小切口采用各种仪器进行高要求手眼配合的手术治疗。由于单孔VATS的视野和器械操作方向类似开胸手术，因此，理论上具有开胸手术经验的高年资外科医师对于掌握单孔VATS是有优势的。

### 3.2

接受过腔镜器械操作技巧，深部操作及器械性能培训胸腔镜外科需要掌握和发展全新的手术技能。相关的手术野信息几乎全靠视觉，有时仅是二维的单角度视觉。利用影像进行手和眼的协调操作必须经过大量训练^[11]^。术者将术野的二维影像转化为大脑中的三维解剖信息，在整个术中二维和三维的解剖认识反复转化和融合，升华到无需思考的生理自然反射反应。

### 3.3 接受过模拟器及动物实验训练

相比模拟器训练，活体麻醉状态下的动物模拟训练，能够提供最佳的模拟手术状态^[12]^。猪是最常用的动物。这是学习训练的最佳方法，但费用较贵，且需要更多的人员配合。

## 4 孔VATS培训模式

单孔VATS技术的培训需要由经验丰富的医生传授手术技巧的同时，传授操作手法背后的提示和诀窍；标准化的手术步骤，树立冷静沉着的心态。这些培训都将有助于增加手术安全性，减少术中并发症和中转率，缩短手术时间，降低术后死亡率^[13]^。

在传统外科技术培训中，带教老师与学者之间的教学实践是建立在患者身上，通过手把手的方式将外科技术直接传授给学生。由于单孔胸腔镜手术操作主要由手术者一个人完成，助手只能从电视屏上观看手术的全过程，很少有动手操作的机会。因此，寻求一种新型的、有效的外科培训模式是十分必要的。根据学者的自身基础，还有培训的长短周期，预期达到的培训效果，我们分别制定不同的培训计划和周期。

### 4.1 短期培训

为了推广胸腔镜外科的发展，一些胸腔镜外科手术开展成熟的医院或胸腔镜生产厂家设立了多种形式的胸腔镜培训中心。培训的方法包括：专家讲课、观看录像、观摩手术、模拟训练、动物实验及跟胸腔镜手术上台体验等。通过培训，能比较系统地了解胸腔镜外科方面的知识，了解胸腔镜设备及器械的功能、操作及维护，初步掌握胸腔镜手术的操作，与有关专家建立初步联系。

#### 4.1.1 自主训练

胸腔镜外科医师培训的另一途径是引进消化，在掌握基本技术后，逐步过渡到临床应用。虽然在培训过程中需投入的精力和时间较多，但在集体办班不成熟时，不失为有效的方法。

#### 4.1.2 视频培训

便捷的网络通讯使得网络视频培训成为可能，外科医生可以通过YouTube或者CTSNet网站，发布一些经典的手术视频，给其他医生提供了网络学习的机会^[13]^。手术视频记录了手术过程中的全部操作，经验丰富的医生可以通过复习回顾，与其他医生讨论手术中的关键步骤，分析错误操作，以此来提高手术技巧^[14]^。

#### 4.1.3 专家会诊指导

要想成为一名合格的胸腔镜外科医师，仅靠短期培训和自主训练显然是不够的。最好能够选择一些合适的病例，请专家帮助逐步开展胸腔镜手术，由助手逐步过渡到术者，再由专家指导独立开展一些简单的手术。

#### 4.1.4 动物实验

模拟训练与临床实际操作尚有差距，用模拟操作箱进行操作，练习者对电刀使用（如切割、电凝止血等）无真实体会。因此，在熟练掌握基本器械操作技术后，进行动物模型练习必不可少，它给练习者提供了类似临床手术的真实环境。

#### 4.1.5 网络手术演示转播

我们在培训基地与手术室之间建立了手术转播系统，可同时转播手术室场景及胸腔镜下操作两套图像，并可在两地之间进行现场双向对话交流。经过上述培训后，学员对胸腔镜技术已经有了较好的了解，此时安排胸腔镜手术技术较娴熟的医师进行胸腔镜手术演示，并在培训现场安排老师进行讲解，对提高学员对胸腔镜手术的了解有较大帮助。

#### 4.1.6 短期-大样本量的临床实践（< 3个月）

在对胸腔镜手术的基本理论、基本技术操作等有了较全面的掌握，可进入临床实践。临床实践通常包括以下几个阶段：①观摩临床手术：这是临床实践的初级阶段，通过观看手术录像、现场观摩手术，进一步体会和感受单孔胸腔镜手术的全过程；②临床扶镜助手阶段：一般先给有丰富胸腔镜手术经验的医师当助手，手术中要仔细理解和体会手术者的每一个操作，手术后细心琢磨，这样才能尽快掌握单孔胸腔镜的技术操作；③临床手术阶段：在完成10次-20次的胸腔镜手术助手、达到合格的要求后，可逐步过渡到手术者。各类胸腔镜手术的学习曲线不同，每个医师的动手能力和灵感也不尽相同，必须要经过长期刻苦的训练，才能逐渐成长为一名合格的临床单孔胸腔镜外科医师^[15]^。

### 4.2 长期培训-临床长期进修学习（> 6个月）

在经过基础理论和胸腔镜技术培训后，若能继续参加临床进修学习，对进一步提高胸腔镜外科技术及独立从事胸腔镜外科均具有重要作用。胸腔镜外科进修选择医院很重要，最好选择已经设立胸腔镜培训中心的医院。我院2017年完成各类胸部手术13, 000例以上，其中胸腔镜手术占80%，单孔胸腔镜占60%以上，成为国内最大的单孔微创胸腔镜中心。胸腔镜外科中心能指定胸腔镜技术熟练的老师负责带教，从手术器械的准备、机器导线的连接，从扶镜者、第一助手到术者逐步过渡。这样既可以系统地接受胸腔镜外科方面的培训，又可以完全融入胸腔镜专业领域的工作，一般来说，通过1年的进修学习，基本能够较熟练掌握胸腔镜操作技巧，并能够独立完成一些常规的胸腔镜手术。

## 5 胸腔镜外科医师的培训内容

不管采取任何一种形式的培训，其培训内容都应该是一致的，即包括基础理论学习、技术训练、临床实践和心理耐压和自信心的培训。具体可从以下几方面入手：

### 5.1

熟练掌握各种微创外科手术的器械和设备的理论基础知识除了熟悉电子显像和传送系统，各种胸腔镜、切割吻合器、超声刀，还有如电凝钩，血管夹、组织牵开器、吸引器等的特性和使用范围，使得在腔镜下各种手术操作都能够得心应手，应用自如。

### 5.2 胸腔镜体外模拟训练

与传统手术一样，胸腔镜手术的基本操作离不开"分离、切割、缝合、打结" 。体外模拟操作训练箱，可以让练习者可在单孔VATS切口的选择，摄像镜头放置、操作器械等，箱体内放置模拟器官（如心脏，主动脉，静脉，肺等）。术者与助手（扶镜者）互相轮换。非生物训练是指单纯的模拟箱内封闭训练，它可以提供在影像下内镜器械的使用练习。练习的内容包括：对靶目标的双手操作，打结练习和离体组织的游离和缝合^[16]^。这些练习虽然缺乏真实感，但费用便宜，易于装配，适合新手基本训练要求。

胸腔镜体外模拟训练应包括以下内容：①手眼协调训练：要求在操作中不可随意碰撞周围，尽量做到稳、准、轻、快。扶镜者应根据手术训练者操作的部位，随时调整镜头及焦距，使术野图像始终保持清晰、准确。左右手协调配合进行，并逐渐加快速度；②定向适应训练：在训练箱内用抓钳将丝线在各个木钉上有目的地进行缠绕，或用丝线完成类似操作；③钳夹和缝合打结训练：要求练习者将剪开的泡沫作间断缝合。首先要求掌握将针线经切口进出操作箱的方法，利用专用持针钳钳夹，进出操作箱时不能脱落离开视野。要掌握进针的深度及针所处位置。反复练习胸腔镜下用操作器械打结的方法，此方法与传统手术中用针持打结方法类似，但缝线不能长；④游离血管，游离支气管/淋巴结操作训练：此部分在单孔VATS训练中是相对较难掌握的部分。学员需要利用各种胸腔内手术器械轻柔，钝性分离不同组织，最终达到游离相应的组织。

## 6 培训标准化与评价

目前还没有统一标准的单孔胸腔镜培训计划，一些有经验的单孔腔镜专家虽然建立一些培训项目，但较难评估培训项目的教育效果，导师水平。然而，我们认为这是一个非常重要的问题，因为单孔VATS是一门具有极高专业性和复杂性的技术。如果缺乏经验的外科医生轻易尝试，可能导致严重的术中意外。因此，培训教学的质量评价理所应当被受到重视和需求。

欧洲胸外科协会出版的VATS培训指南指出：接收培训的VATS人员必须经过先易后难，逐步推进的培训过程。必须完成100台简单基本的VATS操作手术后，才能开始接收大的肺解剖性手术，每年至少完成25台VATS肺叶切除^[17]^。在这样一个循序渐进，逐步成熟的培训过程中，受训者不仅掌握了技术技巧，更加培养锻炼了青年胸外科医生的独立自主和自信心。

对于学习者的分级评价包括：对参加讲座，观摩手术演示，参加动物实验，模拟手术操作，临床扶镜，基本手术操作，独立手术操作的评分评估^[18]^。

## 7 结论

单孔VATS治疗肺癌的可行性已经被多项研究证实^[8]^。由于微创腔镜肺叶切除术同样可以达到彻底根治的目的，正受到越来越多青年医师的青睐。随着单孔VATS在全球快速推广，对于该技术的专业培训需求也与日俱增。

传统的单一的小样本量的培训模式显不能充分满足这样大的需求，一种新由网络视频，手术同步演示，模拟训练，动物实验，高通量手术强化培训，中长期系统性单孔VATS中心进修的培训策略，在培养单孔VATS胸外科医生中扮演了重要角色。未来理想的培训模式或将是标准化的，由政府认可支持，国际专业领域协会保障患者安全性和学术性基础上的综合培训体系。
